# The impact of the exodus of big pharmaceutical companies from Nigeria on antimicrobial resistance in the West African subregion

**DOI:** 10.1186/s44263-024-00068-z

**Published:** 2024-05-24

**Authors:** Chiamaka Norah Ezeagu, Semeeh Akinwale Omoleke, Kehinde Kazeem Kanmodi

**Affiliations:** 1 School of Health and Life Sciences, Teesside University, Middlesbrough, UK; 2 Cephas Health Research Initiative Inc, Ibadan, Nigeria; 3 The Engelhardt School of Global Health and Bioethics, Central African Republic and The Gambia, Euclid University (EUCLID), Banjul, Gambia; 4 School of Dentistry, University of Rwanda, Kigali, Rwanda; 5 Faculty of Dentistry, University of Puthisastra, Phnom Penh, Cambodia

## Abstract

The departure of pharmaceutical companies from Nigeria — a major source of antimicrobial drug supply in West Africa — increases the risk of an elevated burden of antimicrobial resistance (AMR) in Nigeria and the West Africa subregion. Urgent actions must be taken by relevant actors to address the increased risk of AMR.

## Background

The exit of GlaxoSmithKline (GSK) and Sanofi from Nigeria could potentiate the risk of antimicrobial resistance (AMR) in the country and West Africa subregion [1]. AMR emerges when a once-effective antimicrobial substance loses its ability to combat susceptible microorganisms [2]. The dominance of communicable diseases, such as malaria, HIV/AIDS, diarrheal diseases, and tuberculosis (TB), in West Africa requires the use of antimicrobials for curative purposes [3]. Nigeria remains a major source of antibiotic supply to other West African countries. However, the availability of these drugs is currently being jeopardized by the exodus of these big pharmaceutical companies.

Further, the big pharmaceutical companies play significant roles in antibiotics development and marketing and implicitly the minimization of AMR. In conjunction with regulatory authorities, they support actions towards the appropriate use of antibiotics, bolstering surveillance, infection control practices, research and development (R & D), and enhancing education on antibiotics use [4]. Consequently, the exodus of these big pharmaceutical companies may likely result in a decline in access to quality drugs and an increase in the sales of substandard antibiotics across the subregion, with an attendant AMR increase.

## AMR situation in Nigeria and West Africa

Nigeria, like other low-and-middle-income countries (LMICs), remains challenged by diseases of poverty, inadequate water and sanitation facilities, and over-stretched hospital services characterized by inadequate infection prevention and control. These factors are relevant to microbial spread and control of infectious diseases, including nosocomial infection. Given the higher rates of communicable diseases in the face of a gradual rise in noncommunicable diseases (NCDs), there is a huge use and misuse of antimicrobials in formal and informal care settings in Nigeria and other West African countries. Specifically, Nigeria has a disproportionately higher rate of antibiotic utilization within the subregion despite weak antibiotic governance and stewardship [4].

Most antibiotics can be accessed over the counter — without prescription — in Nigeria and across the subregion [4]. This enhances irrational use in the form of underdose, incomplete dosage, or outright consumption of substandard medication leading to the development of resistant pathogens after exposure to nonlethal doses. Further, weak regulatory capacity, unrestricted access, poor awareness about AMR, and lack of formal antibiotics stewardship in most formal health institutions also provide a fertile environment for the AMR epidemic in Nigeria [4, 5] and possibly within the subregion. The issue of AMR is predominant here and is influenced in part by inappropriate use of antimicrobial agents [4]. In West Africa, the burden of NCDs keeps increasing, and a rising AMR burden might be contributory. AMR is currently undermining the treatment of prevalent infections, such as respiratory infections (e.g., TB), diarrheal diseases (e.g., cholera, dysentery), and sexually transmitted infections (e.g., HIV), and the prevention of NCDs caused by carcinogenic pathogens (as seen in cervical, colorectal, and gall bladder cancers).

## The exodus of big pharmaceutical companies

In August 2023, GSK announced the decision to withdraw from Nigeria due to operational difficulties, bringing an end to its presence in the country after 51 years [1]. Shortly after GSK’s resolve, Sanofi, renowned for polio vaccine production, announced exodus plans [1]. To ensure the continuous availability of their products in the country, GSK and Sanofi have transitioned to a third-party distribution model [1]. This third-party distribution company would be responsible for receiving, storing, and delivering their products to healthcare facilities, pharmacies, and other points of sale.

One of the challenges faced by the big pharmaceutical companies resulting in their exit from the country is recurrent foreign exchange instability [1]. The nonavailability of foreign exchange had an impact on their capacity to fulfill trade debts in foreign currency with suppliers of input goods, creating difficulties in maintaining a consistent supply to the market [1, 6]. This resulted in the outsourcing of the supply chain to a third party. Although these reasons are logical and valid, their departure would constitute a huge gap in the pharmaceutical delivery of products and services in Nigeria with a spillover effect in the subregion. This has huge implications on the product availability, pricing, and affordability.

## Implications for AMR in West Africa

The implications of the exodus of the big pharmaceutical companies on AMR and the broader public health consequences constitute a significant threat to communicable disease and NCD control.

First, the reliability and safety of pharmaceuticals, and resistance-preventing healthcare tools like vaccines and diagnostics, hinge on the strength and security of their supply chain [7]. The exodus of the big pharmaceutical companies from Nigeria is poised to disrupt the drug supply chain across West Africa and beyond, potentially exacerbating AMR in the subregion. The absence of these key players has led to shortages of essential antimicrobial medications, forcing healthcare providers to resort to alternatives due to high costs, and potentially less effective treatments. Such disruptions contribute to inappropriate antibiotic use and the emergence of drug-resistant pathogens [7]. Consequently, the healthcare systems in the subregion may face greater challenges in effectively treating common infections and infestations, ultimately impacting patient outcomes and public health efforts to control infectious diseases.

Additionally, restriction on the availability of quality drugs potentially abounds. Quality medications are highly necessary for effectively treating infections and curbing the development of resistance. With the exit of these big pharmaceutical companies from the Nigerian market, there is a real threat of compromised access to essential antibiotics and related medications in West Africa. Studies have highlighted the detrimental effects of substandard and counterfeit drugs on the efficacy of antimicrobials, leading to the emergence and spread of resistance [8]. The absence of quality antimicrobials from such reputable companies may pave way for the proliferation of inferior medications, further fueling the AMR crisis [8], given the limited national regulatory capacities.

Regarding the impact on NCDs, the disease control efforts in the subregion would be impacted negatively. For instance, Cervarix vaccine produced and widely distributed by GSK for the prevention of anogenital lesions would most likely be inaccessible. This would cause a local resurgence of human papillomavirus-related cancers. Shortages in drugs and vaccines might necessitate changes in treatment approaches and difficulties in patient compliance. This is in addition to a potential hike in the cost of drugs and antibiotics [9].

Finally, the exodus of the big pharmaceutical companies would adversely impact R&D, leading to reduced efforts in combating AMR in Nigeria and the West African subregion. R&D initiatives play a pivotal role in discovering and developing new antimicrobial agents, as well as enhancing existing treatment options and promoting antimicrobial stewardship practices [3]. The absence of GSK and Sanofi in Nigeria diminishes the country’s and regional capacities for innovation and R&D investment in antimicrobial drugs and vaccines, which are essential in addressing emerging resistance [3]. Reduced investment in R&D could lead to stagnation in the development of novel antimicrobial therapies, leaving healthcare providers with limited options to combat resistant infections and contributing to the further proliferation of AMR. Thus, the departure of these companies not only threatens access to essential medications but also undermines efforts to develop newer and effective tools against antimicrobial resistance in West Africa.

## Actions to mitigate the risks

To tackle the risk of antimicrobial scarcity, it becomes a necessity to enhance production of pharmaceuticals by local companies [1]. Offering incentives to encourage local manufacturing and simplifying regulatory processes may prove instrumental in achieving this goal. Collaboration between the Nigerian government and global pharmaceutical companies could ensure a consistent provision of vital medications. Vigilant supervision and meticulous inventory control are imperative to prevent medication deficits. Regulatory authorities should expedite the approval process for alternative suppliers, while healthcare providers need to actively manage medication inventories to mitigate gaps in the supply chain [6].

Moreover, antimicrobial stewardship programs (ASPs) are integral in the global fight against AMR, implemented across various sectors and nations with proven successes [10]. The execution of ASPs entails initiatives aimed at fostering responsible antibiotic usage both within healthcare facilities and in broader settings. Key components of hospital ASPs involve establishing dedicated leadership accountable for program effectiveness and striving to enhance antibiotic administration practices. Additional critical aspects encompass the adoption of recommended strategies, continual monitoring of antibiotic prescriptions aligning with established guidelines and current resistance trends, regular dissemination of information to all relevant stakeholders, and educating prescribers on resistance dynamics and optimal prescribing practices. Reported advantages of ASPs include enhanced quality and safety of patient care, more effective treatment of infections, curbed dissemination of AMR, decreased antibiotic consumption, minimized adverse effects associated with antibiotics, and lowered healthcare expenditures [10].

## Conclusions

The departure of pharmaceutical companies from Nigeria threatens local and regional public health, potentially exacerbating AMR. The real challenges include drug shortages, rising costs, compromised quality, and potentially reduced research efforts. Urgent actions are needed, such as promoting local production, reducing import duties on pharmaceutical equipment and inputs, fostering collaboration, and implementing robust regulatory oversight. ASPs are indeed crucial. By prioritizing access to quality medications, responsible antibiotic use, and research investment, Nigeria and West African subregion can mitigate the risks posed by the exodus of the big pharmaceutical companies and safeguard public health against the growing threat of AMR.

## Data Availability

No datasets were generated or analysed during the current study.
